# Genome-wide functional genomic and transcriptomic analyses for genes regulating sensitivity to vorinostat

**DOI:** 10.1038/sdata.2014.17

**Published:** 2014-07-08

**Authors:** Katrina J Falkenberg, Cathryn M Gould, Ricky W Johnstone, Kaylene J Simpson

**Affiliations:** 1 Cancer Therapeutic Program, The Peter MacCallum Cancer Centre, St Andrews Place, East Melbourne, Victoria 3002, Australia; 2 Victorian Centre for Functional Genomics, The Peter MacCallum Cancer Centre, St Andrews Place, East Melbourne, Victoria 3002, Australia; 3 Sir Peter MacCallum Department of Oncology, The University of Melbourne, Parkville, Victoria 3052, Australia

## Abstract

Identification of mechanisms of resistance to histone deacetylase inhibitors, such as vorinostat, is important in order to utilise these anticancer compounds more efficiently in the clinic. Here, we present a dataset containing multiple tiers of stringent siRNA screening for genes that when knocked down conferred sensitivity to vorinostat-induced cell death. We also present data from a miRNA overexpression screen for miRNAs contributing to vorinostat sensitivity. Furthermore, we provide transcriptomic analysis using massively parallel sequencing upon knockdown of 14 validated vorinostat-resistance genes. These datasets are suitable for analysis of genes and miRNAs involved in cell death in the presence and absence of vorinostat as well as computational biology approaches to identify gene regulatory networks.

## Background & Summary

Histone deacetylase inhibitors (HDACi) are a novel class of anti-cancer agents that elicit a range of anti-tumour responses including apoptosis. Vorinostat is an FDA-approved broad-spectrum HDACi, which has achieved remarkable clinical success in some patients^1,2^, particularly those with Cutaneous T cell lymphoma and Peripheral T cell lymphoma, however it remains unclear why certain patients remain unresponsive. Constitutive STAT activation^3^, overexpression of pro-survival Bcl-2 proteins^4^ and loss of HR23B^5,6^ have been identified as potential biomarkers of HDACi resistance, however none have improved the clinical utility of HDACi. High-throughput RNAi screening has demonstrated utility for identification of novel drug targets for cancer therapy and biomarkers for drug response^7–10,7–10,7–10,7–10^. In addition, screening for synthetic lethality has successfully identified targetable cancer-specific vulnerabilities alone and in combination with existing therapies^11–15,11–15,11–15,11–15,11–15^. Therefore, the study described within aimed to further elucidate vorinostat resistance mechanisms through a functional genomics screen to identify genes that when knocked down by RNA interference (RNAi) sensitised cells to vorinostat-induced apoptosis. Specifically we aimed to identify vorinostat-resistance genes that had not previously been described as important in vorinostat response. These genes may serve as molecular biomarkers for stratification of patients for HDACi treatment or as potential novel drug targets for development of new therapies to be used in combination with vorinostat.

A synthetic lethal functional screen using a protein-coding genome-wide RNAi library was used to identify genes that when knocked down co-operated with vorinostat to induce tumour cell apoptosis in otherwise resistant cells. Briefly, cells were reverse transfected on day 1, media changed on day 2, treated with vorinostat or vehicle (DMSO) on day 3 and assessed for cell death on day 4. The screen contained two arms: the ‘minus-drug’ (DMSO control) arm to determine those genes that were lethal by gene knockdown alone and would therefore be excluded from the analysis and the ‘plus-drug’ (vorinostat treatment) arm to identify those genes that when knocked down, co-operated with vorinostat to induce cell death. The two parallel arms of the screen evaluated cell death using different measurements. The plus-drug arm was evaluated for general viability using Cell Titre Fluor (CTF) and apoptosis using Caspase-Glo 3/7. Together these assays constitute the Apo-Live Glo multiplexed assay (Promega). The multiplex assay was necessary to be able to distinguish rapid cell death from slower activation of caspase activity within the window of the assay and meant that different stages of cell death could be identified in the same sample at a single time point. The minus-drug arm was subjected to nuclear staining by DAPI followed by high content cell counting as a surrogate readout of cell death, thereby reducing cost and facilitating detection of strong cell cycle effects. Supplementary Figure 1 shows there was a high correlation between CTF, cell counting and Caspase 3/7 activity.

**Figure 1:**
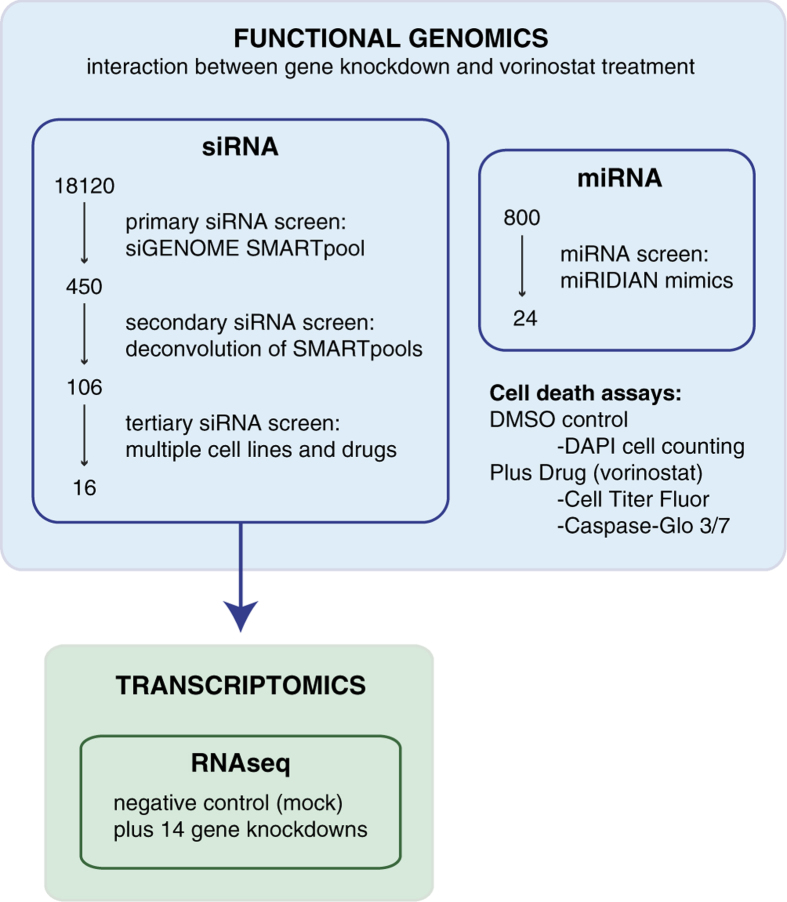
Experimental workflow. This study consisted of functional genomics and transcriptomics approaches. A tiered siRNA screening approach is depicted in tandem with a miRNA overexpression screen. Transcriptional profiling was conducted on 14 candidates resulting from the siRNA screen.

The primary SMARTpool siRNA screen yielded 450 gene hits, of which 106 validated in a secondary deconvolution screen using the four individual constituent siRNAs of each SMARTpool. Tertiary screening was conducted to evaluate the specificity of these genes to co-operate with vorinostat compared to conventional chemotherapeutics in multiple cell lines. Comparative gene expression analysis was undertaken upon knockdown of 13 vorinostat-resistance candidates from the tertiary siRNA screen. In addition, a miRNA mimic (overexpression) screen was conducted to identify miRNA involved in vorinostat sensitivity. Figure 1 is a graphical representation of the experimental design employed in this study. The RNAi datasets described within will be useful for analyses of protein-coding genes and miRNAs crucial to cell survival in steady state and in the context of HDACi treatment. The gene expression profiling datasets will be suitable for computational biology analysis as they represent transcriptional changes in a cell line subjected to different perturbations (i.e., gene knockdown).

## Methods

### Cell culture

Four human colon cancer cell lines were used in this study: HCT116-VR, SW480, SW620, LIM1215. HCT116-VR were obtained from Merck Sharp and Dohme and were generated by culturing parental HCT116 cells in increasing concentrations of vorinostat, thereby obtaining a vorinostat resistant (VR) line. All cell lines were cultured in humidified incubators at 37 °C, 5% CO_2_. Refer to Table 1 for media and transfection conditions for each cell line.

### High throughput RNA interference screening

#### Overview of RNAi screening methodology

The RNAi screen was performed in 384 well plate format in technical duplicate. For each library plate, four assay plates were transfected. In the case of pooled siRNAs (primary and tertiary screens) the siRNA concentration was 40 nM, while individual sequences were screened at 25 nM (deconvolution screen), as per standard practice in the field. Replicate plus-drug plates were termed ‘A’ and ‘B’ and replicate minus-drug plates were termed ‘C’ and ‘D’. Positive and negative controls were positioned in columns 2 and 23 (eight wells for each control) and media only in column 24 of each assay plate. The positive controls were technical (PLK1 for cell death; doubled as a positive control for CTF) and assay specific (JAK2 for Caspase-Glo 3/7). The negative control was mock transfection (lipid only). At the time the screen was conducted we could not identify a non-targeting control that exhibited no phenotype. All liquid dispensing steps were carried out with the EL406 Microplate Washer Dispenser (BioTek) except the transfection itself, which required the SciClone ALH3000 Lab Automation Liquid Handler (Caliper Lifesciences). The plate reader used was the Synergy H4 Hybrid Multi-mode Microplate Reader plate reader (BioTek).

#### Screening reagents

- Dharmacon siGENOME siRNA library RefSeq27
- Dharmacon miRIDIAN miRNA mimic library miRbase13
- DharmaFECT 2 (Thermo Scientific, cat # T2002-03)
- opti-MEM (Life Technologies, cat # 51985091)
- Poly-L-lysine, 0.01% solution cell culture tested (Sigma, cat # P4707)
- Apo-Live Glo multiplexed assay (Promega, cat # G6411)
- Cell Titre Fluor, extra (Promega, cat # G6082) (note: additional reagent was required due to diluting the caspase component of the Apo-Live Glo kit to half that of the manufacturer’s recommendation)
- 4′,6-diamidino-2-phenylindole (DAPI) (Invitrogen cat # D8417) 5 mg/ml
- 4% paraformaldehyde (PFA) in Tris (16% PFA pre-made solution (Electron Microscopy Sciences, cat # 15710) diluted one part 16% PFA to 3 parts Tris (50 mM pH 7.5))
- 50 mM Tris pH7.5, filtered
- Triton-X-100, 10% in H_2_O

#### Poly-L-lysine plate coating

HCT116-VR cells were weakly adherent at the early stages of transfection when sparse and liquid-handling conditions exacerbated the problem. Therefore a poly-L-lysine plate coating was used to improve adherence. 384-well plates were coated with poly-L-lysine (MW 70,000–150,000) the week prior to plate use. Poly-L-lysine was diluted 1:8 in PBS, 20 μl dispensed to each well and plates incubated for 1 h at room temperature. Poly-L-lysine solution was removed, wells rinsed twice with 50 μl PBS and air dried prior to storing at 4C.

#### Day 1: Reverse transfection

siRNA library and control plates were thawed at room temperature 1 h prior to use. Cells were incubated in TrypLE (Life Technologies) for three minutes, washed in growth media, counted and diluted to the desired concentration (Table 1). DharmaFECT 2 was diluted in Opti-MEM at the appropriate concentration and incubated for 5 min before dispensing 44 μl (4x volume) into the representative A plate for each siRNA library plate (Biotek). For the primary screen, 6 μl of the siRNA library (4x volume of 1 μM library) was dispensed into the lipid/Opti-MEM (SciClone), mixed and distributed in independent liquid handling steps (returning to the A plate each time) to the B, C and D plates (12.5 μl each), thus ensuring each assay plate was derived from the same transfection mix. Plates were incubated for 20 min at room temperature before dispensing 25 μl cells to each well (Biotek) (total volume 37.5 μl: 12.5 μl siRNA/DharmaFECT 2/Opti-MEM plus 25 μl cells). Column 24 received media only (no cells) for background fluorescence and luminescence readings.

#### Day 2: Media change

Media was changed on all assay plates 24 h after transfection. A high aspirate setting leaving ~10 μl/well was used to ensure cells remained attached to the plate. In order to overcome any toxicity that may arise from residual transfection lipid remaining in the wells, a large volume of pre-warmed fresh growth medium (50 μl) was added to each well. Plates were incubated for a further 24 h.

#### Day 3: Drug treatment

Media was removed from wells as above and replaced with 15 μl of drug- or vehicle-containing growth medium. Cells were treated with vorinostat at a concentration of 4.17 μM to achieve a final concentration of 2.5 μM. Vehicle treated wells received medium containing an equivalent volume of DMSO. Etoposide treated wells received 8.33 μM etoposide to achieve a final concentration of 5 μM (tertiary screen only). Plates were incubated for a further 24 h.

#### Day 4: Assays (Apo-Live Glo and nuclear staining)

The assays discussed below have been described in detail in Falkenberg *et al.*, ‘A High-Throughput, Multiplex Cell Death Assay Using an RNAi Screening Approach’^16^ and the reader is directed to this article for detailed, step by step instructions. A summary of the steps involved in each assay is provided below. In brief, the Apo-Live Glo multiplexed assay used for the plus-drug arm first measures cell viability using the fluorescent Cell Titer Fluor reagent, then caspase activation *via* cell lysis and luminescent readout of Caspase 3/7 activity using the Caspase-Glo 3/7 reagent.

*Cell Titre Fluor*: CTF is a viability assay, whereby a fluorescent signal is generated in live cells through live-cell protease cleavage of a fluorogenic peptide substrate. The fluorescent signal is proportional to the number of live cells with intact plasma membranes. CTF substrate and buffer were combined in the ratio of 10 μl substrate to every 2.5 ml buffer. 5 μl reagent was added to each well of the A and B plates, plates were placed on a microtiter plate shaker (MPS1, Ratek) at half maximum speed for one minute and incubated at 37 °C in a humidified incubator for 1.5 h. Fluorescence was detected with excitation: 380–400 nm and emission: 505 nm (Synergy).

*Caspase-Glo 3/7*: Caspase 3/7 Glo measures the amount of activated caspases 3 and 7 within each well using a luminogenic caspase 3/7 substrate. The reagent lyses cells, allowing cleavage of the substrate into the mature form, which is a luciferase substrate. Cell lysis, substrate cleavage and the luciferase reaction occur to produce light, which is proportional to the amount of activated caspases in the sample. Caspase-Glo 3/7 lyophilised substrate and buffer were combined and 12 μl dispensed to each well of the A and B plates. Plates were placed on a microtiter plate shaker (MPS1, Ratek) at half maximum speed for one minute and incubated for 30 min at room temperature followed by luminescence detection (Synergy).

*Nuclear staining for cell counting*: DAPI nuclear staining was used to label each cell on the C and D plates for cell counting. First, medium was aspirated leaving 15 μl and cells were fixed by addition of 15 μl 4% PFA (final concentration 2% PFA). Plates were incubated for 10 min, before removal of PFA and rinsing with Tris. Cells were permeabilised and stained with DAPI in a single step by adding DAPI solution containing Triton-X-100 (final concentration: DAPI 5 μg/ml, Triton-X-100 0.2% (v/v)). Cells were incubated for 15 min before removal of stain and addition of 50 μl PBS per well. Plates were imaged on the Cellomics ArrayScan (Thermo Scientific) high content imager. The Cellomics Cell Cycle proprietary algorithm was optimised for object segmentation and was instructed to count a maximum of 1,500 cells or 25 fields, whichever was reached sooner. These cut-offs were based on negative controls, which consistently counted ≥1,500 cells in 7-8 fields.

#### Data analysis

*Quantitation of cell number*: A binning strategy incorporating cell count and field number was defined to evaluate the health of each well (SA=single agent). This allowed identification of wells with large amounts of cell death in the minus-drug arm that could be removed from downstream analysis in the plus-drug arm. Pairs of duplicate plates were averaged prior to binning.

*Primary siRNA screen and miRNA screen*

SA1 - Toxic bin: <1,500 cells counted in 25 fields

SA2 - Very healthy bin: ≥1,500 cells counted in ≤11 fields

SA3 - Moderately healthy bin: all other wells (≥1,500 cells counted in 12–24 fields, inclusive)

*Secondary and tertiary siRNA screens*

SA1 - Toxic bin: <1,500 cells counted in 25 fields

SA2 - Healthy bin: ≥1,500 cells counted in ≤25 fields

*Caspase-Glo 3/7 and Cell Titre Fluor*: The media only column on each plate was averaged and subtracted from all other values on that plate. For the primary siRNA and miRNA screens, Caspase-Glo 3/7 and CTF data was normalised on a per plate basis using fold change to the mock transfection negative control (negative control normalisation). The robust *z*-score^17^ was then applied as a hit identification strategy across all screen plates based on average fold change outcome per set of duplicate plates. For the deconvolution and tertiary screens, this additional sample-based normalisation across all plates was not appropriate so fold change to the negative control (mock) was used for each sample. Hits were binned according to the following criteria (RV=reduced viability, CA=caspase activation, NC=no change).

*Primary siRNA screen and miRNA screen*

RV1: SA2 and reduced viability     CA1: SA2 and caspase activation

RV2: SA3 and reduced viability     CA2: SA3 and caspase activation

NC: all other wells           NC: all other wells

*Secondary and tertiary siRNA screens*

RV: SA2 and reduced viability     CA: SA2 and caspase activation

NC: all other wells           NC: all other wells

*Seed cluster analysis*: As siRNA-based RNAi technology is modeled on the cell endogenous process, seed sequence analysis was conducted (Dharmacon Thermo Scientific Bioinformatics service) to determine the likelihood of particular siRNAs acting as an endogenous miRNA. This analysis was conducted after completion of the primary screen. The list of the 450 top scoring genes and 900 genes that were the closest to having no effect (i.e., robust *z*-score close to zero, null effect, or ‘nulls’) were analysed for over-representation of seed sequences in the hits compared to the nulls and endogenous miRNAs mapped to these seed sequences if known. As miRNA seed prediction did not demonstrate miRNA-like off-target effects of the top ranking 450 genes, this complete gene list was taken for further investigation through the deconvolution validation screen.

### Description of tiered RNAi screening

#### Primary genome-wide siRNA screen

**Figure 2:**
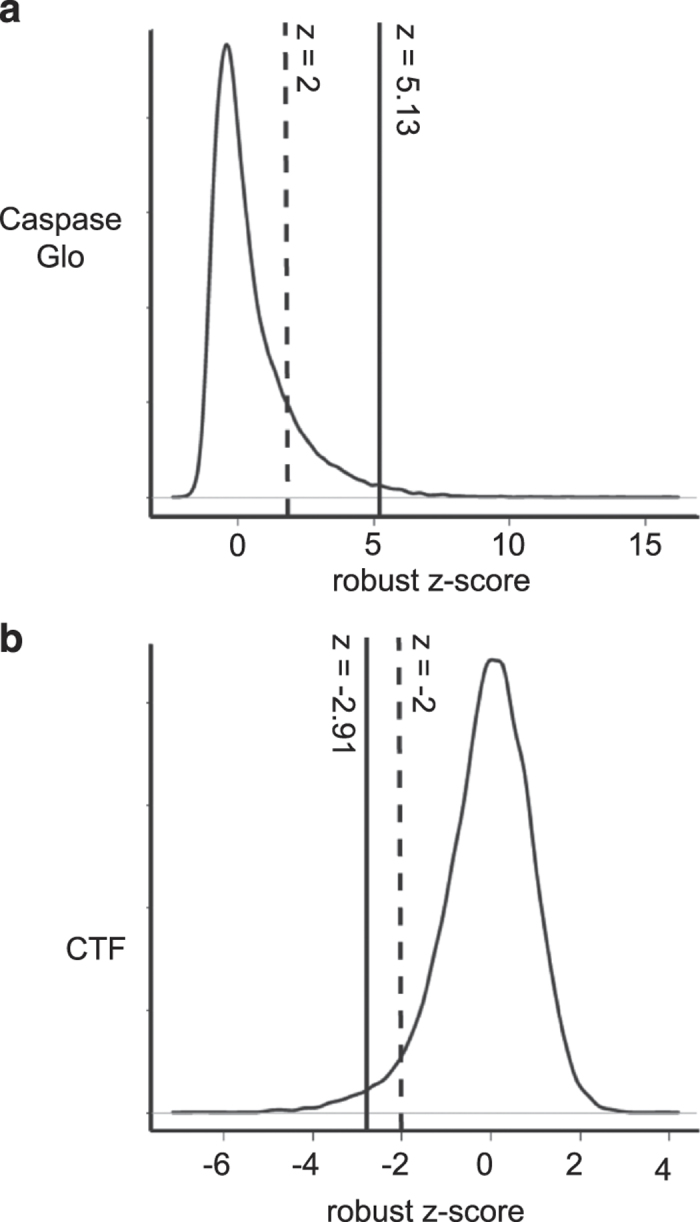
Caspase-Glo 3/7 and CTF data distributions for the primary siRNA screen. Plots showing the genome-wide robust *z*-score distribution for (**a**) Caspase-Glo 3/7 and (**b**) CTF. The data shown is the complete screen data excluding controls and lethal wells identified in the DMSO control DAPI stained, cell-counting arm. These plots display the distribution of robust *z*-scores (X axis) in terms of smoothened frequency (Y axis) for the primary siRNA screen. These curves do not reflect a normal distribution of hits, as the screen was set up to sensitise for cell death. Note that in the Caspase-Glo 3/7 data (**a**) (and to a lesser extent, the CTF data (**b**)), a robust *z*-score cut-off of +2 or −2 (dotted lines) would have resulted in far too many hits to reasonably follow up (several thousand). Instead a cut-off of *z*≥5.13 was used for Caspase-Glo 3/7 and *z*≤2.91 for CTF (solid lines), to give a total of 450 primary screen hits. siJAK2, the moderate strength positive control for Caspase 3/7 activity had a robust *z*-score of 2.24 and siPLK1, the positive cell death control for CTF had a robust z-score of −4.48.

*Analysis*: After the completion of the primary screen, minus-drug DAPI stained plates were used to determine that 191 genes were lethal by gene knockdown alone. For Caspase-Glo 3/7 and CTF, all plus-drug plates were analysed as an entire screen. Average background fluorescence and luminescence was removed on a per plate basis, followed by normalisation to the mock negative control. Duplicates plates were averaged and robust *z*-score normalisation applied across the entire screen. Following removal of controls and the 191 genes whose knockdown alone was lethal, Caspase-Glo 3/7 and CTF data was plotted in smoothed histograms showing the overall shape of the data distribution (Figure 2). With robust *z*-score plotted against relative abundance, it was clear that Caspase-Glo 3/7 data was strongly positively skewed with a long tail of high robust *z*-scores (Figure 2a). The distribution of CTF was slightly negatively skewed (Figure 2b). The robust *z*-score cut-offs were chosen to reflect the nature of the experimental design with approximately two thirds Caspase-Glo 3/7 hits and one third CTF hits as the screen was primarily set up to detect induction of apoptosis, with the viability readout as an extra measure so as not to omit very fasting-acting hits. Robust *z*-score cut-offs of *z*≥5.13 for Caspase-Glo 3/7 and *z*≤ −2.19 for CTF were chosen (originating from both SA2 and SA3 DAPI designations), resulting in a gene list of 450 targets, comprising 150 CTF (reduced viability) hits and 317 Caspase-Glo 3/7 (caspase activation) hits, with an overlap between the two assays of 17 genes (Data record 1). The chosen discovery rate of 450 targets represents ~2.5% of the genome. This cut off number met the criteria defined by the screening facility to be included within the cost of a screen.

#### Secondary deconvolution siRNA screen

*Methodology*: The standard practice in pooled siRNA screening is to conduct a deconvolution screen, where each of the individual siRNA sequences of the top scoring hits are screened individually to provide a technical validation of the reagents^18–20,18–20,18–20^. The 450 top scoring genes from the primary screen were assessed in a secondary deconvolution screen, where all four siRNA sequences per gene were on the same library plate. 32 negative control (mock) wells were included on every plate for control-based normalisation. Positive controls (JAK2 and PLK1) were maintained at 40 nM with 8 wells per plate. Controls were positioned in columns 7, 12, 17 and 22. Targets were excluded from the outer 2 rows and columns (Supplementary Figure 2).

*Analysis*: The primary screen hits were determined by a robust *z*-score cut-off, however a *z*-score is inappropriate for the analysis of duplexes by the inherent nature of them all being hits. We used fold change to mock to validate the duplexes. The cut off thresholds were initially set based on the fold change value associated with the primary screen bin robust *z*-score cut off resulting in a value of 2.58 for Caspase-Glo 3/7 and 0.56 for CTF. However, the dynamic range of the secondary screen was reduced compared to the primary screen in terms of both controls and samples (average 31% less Caspase-Glo 3/7 luminescence and 12% less CTF fluorescence). This may have been due to higher raw values for mock in the secondary screen compared to the primary screen for both Caspase-Glo 3/7 (average 11% more luminescence) and CTF (average 23% more fluorescence) and batch variation of the assay reagents. A gene was considered to be validated with high confidence if 3 or 4 of the 4 duplexes reproduced the SMARTpool phenotype (i.e., 3/4 and 4/4). Given the reduced dynamic range, we re-assessed our hit list and looked more closely at targets initially validating as 3/4 and 4/4 based on the primary screen fold change cut-offs. We then revised our cut-offs based on the mean and standard deviation of fold change to mock for these genes and determined new fold change cut-offs as the mean minus or plus one standard deviation for Caspase-Glo 3/7 and CTF respectively. As a result, the secondary screen fold change cut-offs were reduced to 2.2 for Caspase-Glo 3/7 and 0.6 for CTF.

Targets for which 2 of the 4 duplexes validated and the remaining 2 duplexes had no phenotype were considered moderate confidence hits and were investigated for the purposes of bioinformatics and pathway analysis. Upon manual observation and literature searching, 14 genes that validated as 2/4 were up-graded into the high confidence gene list, as they were members of gene families or pathways of interest, with a third duplex failing to meet validation cut-offs by only a narrow margin. These genes were: SAS10, NPCL1, M96, SEMA4F, RERE, CBX7, CCNK, MED28, POLR2J, CDK10, EIF2B2, EIF2S1, EIF3S6IP, HIST1H1B. In total, the final validated gene list consisted of 106 genes: 31 scored 4/4, 61 scored 3/4 and 14 scored 2/4 (Data record 2). 11 of these genes validated with high confidence in both assays. The two screening assays measured different stages of cell death so the majority of hits would be expected to be an exclusive hit in one assay or the other, however having high caspase activity (Caspase-Glo 3/7) is not mutually exclusive of a reduction in viability (CTF).

#### Tertiary siRNA screen with multiple cell lines and drugs

*Methodology*: To triage the 106 high confidence genes further, a tertiary screen was performed in which a panel of colorectal cancer cell lines (HCT116-VR, SW480, SW620, LIM1215) were treated with vorinostat and the conventional chemotherapeutic, etoposide. The aim was to identify genes that when knocked down sensitised specifically to vorinostat and not etoposide in multiple cell lines to reduce the list of candidates to those that acted specifically in concert with HDAC inhibition. The tertiary screen followed the same basic experimental workflow as the primary screen for each of the 4 cell lines, transfected in parallel. The tertiary screen library of 106 target SMARTpools was arrayed twice side-by-side on the library plate, allowing each half assay plate to be treated with a different drug. The plus-drug plates received vorinostat on one half and etoposide on the other. The minus-drug plates received DMSO.

*Analysis*: New fold change cut-offs were defined for the tertiary screen to reflect the challenges of screening in multiple cell lines with different responsiveness to the combined effect of gene knockdown and drug treatment. The thresholds from the primary and secondary screens were lowered in accordance to reduced dynamic range of the tertiary screen while still maintaining selectivity for enhanced cell death. A fold change to mock of 0.75 or less was defined as the CTF cut-off and 1.6 or greater as the Caspase-Glo 3/7 cut-off. Each of the four cell lines was assessed in terms of CTF and Caspase-Glo 3/7 for vorinostat and etoposide treatment. For each assay (CTF and Caspase-Glo 3/7) the number of cell lines exceeding the defined threshold upon vorinostat treatment and etoposide treatment were counted. To be considered a validated, vorinostat-specific target, a gene must have scored as a hit in at least 2 more cell lines for vorinostat than etoposide. Of the 106 genes tested, 16 genes exhibited selectivity to vorinostat in multiple cell lines according to the criteria above (Data record 3). 15 of these genes were hits from the Caspase-Glo 3/7 assay and a single gene was a hit from the CTF assay.

#### miRNA overexpression (mimic) screen

The Dharmacon miRIDIAN miRNA mimic library version 13 contained 879 synthetic miRNAs (miRNA mimics) arrayed in 384 well format. The experimental workflow was the same as for the primary siRNA screen. Using the same statistical analyses and thresholds determined in the primary siRNA screen, a list of 24 miRNA hits was generated (Data record 4). In summary, there were 10 Caspase-Glo 3/7 hits and 14 CTF hits and no overlap between the two assays.

### Transcriptome analysis using next generation sequencing

#### Preparation of RNA for NGS

Transcriptome analysis was conducted on HCT116-VR cells subjected to gene knockdown using Next Generation Sequencing (NGS) technology. 13 of the 16 genes from the tertiary screen were evaluated, as well as the screening control, *JAK2*. RNAseq was conducted on libraries generated from three independent biological experiments. HCT116-VR cells (2×10^5^ cells per well) were reverse transfected in 12 well plates and harvested after 48 h for RNA extraction. Total RNA was extracted using the RNeasy Plus Mini Kit (Qiagen) as per manufacturer’s instructions. RNA concentration and purity was assessed by spectrophotometry (Nanodrop) and RNA integrity was assessed using the Agilent RNA Nano chip on the Agilent Technologies 2100 Bioanalyzer. Samples with a concentration of at least 170 ng/μl, a 260/280 ratio between 2.0–2.3 and RNA integrity number (RIN) of at least 7.0 were accepted for library preparation.

#### Preparation of samples for the Illumina HighSeq 2000

RNA was prepared for sequencing on the Illumina HighSeq 2000 using the proprietary *TruSeq*TM *RNA Sample Preparation v2 Guide* with all kit reagents purchased from Illumina. This method was chosen as it is very robust and involves poly-A selection for enrichment of mRNA. In brief, poly-A containing mRNA was purified from 2 μg total RNA using poly-T oligo-attached magnetic beads in two rounds of purification. This was followed by RNA fragmentation and first strand cDNA synthesis with reverse transcriptase and random hexamer primers. The RNA template was removed and a replacement strand synthesised to generate double-stranded (ds) cDNA. After purifying the ds cDNA from the reaction mix with AMPure XP beads (Beckman Coulter), the Illumina *End Repair* strategy removed overhangs and prepared the ds cDNA for adapter ligation in a way that prevented concatenation of fragments. Indexing adapters were ligated to the ends of the ds cDNA, which enabled hybridisation to the flow cell and multiplexing of samples within the flow cell. Next, the ds cDNA libraries were PCR amplified to enrich for fragments with adapters on both ends and to amplify the amount of DNA in the libraries. DNA concentration was assessed using the Qubit (Invitrogen) and average fragment size and purity was determined using the Agilent DNA-1000 chip on the Agilent Technologies 2100 Bioanalyzer. Samples of concentration 25–40 ng/μl and 290–340 bp in size were accepted for sequencing. Final determination of concentration was achieved by qPCR according to the Illumina *Sequencing Library qPCR Quantification Guide*. This was followed by normalisation to 10 nM and pooling of libraries, followed by single end sequencing on the Illumina HighSeq 2000 (Data record 5).

#### NGS sequence alignment and differential gene expression analysis

RNAseq reads were aligned to the human reference genome (Homo_sapiens.GRCh37.67.gtf (from ENSEMBL release 67)) using TopHat/Bowtie^21^. Sample quality control was performed simultaneously with the FastQC program (Babraham Bioinformatics, Babraham Institute). Read counting was performed with HTSeq-Count from HTSeq^22^. Non-reduction of the expression matrices was employed to output counts for all Ensembl gene IDs. The Integrative Genomics Viewer (IGV) browser (Broad Institute) was used to visualise the alignment of RNAseq reads against the reference genome. Differential gene expression analysis was performed using EdgeR^23^ and Limma-Voom^24,25^. The Limma-Voom package was used for data normalisation, to account for different numbers of reads per sample, and generation of differential expression gene matrices. All siRNA knockdowns were compared to mock transfection. Expression matrices generated were the raw expression matrix (containing raw read counts), normalised expression matrix (containing log_2_ expression values) (Data record 5) and differential expression matrix (containing log_2_ fold changes).

## Data Records

### Data record 1

Primary siRNA screen data are available at PubChem under the accession number AID 743454 (Data Citation 1). Screen-wide raw and normalised data (negative control normalisation and robust *z*-score normalisation, where appropriate) are provided for the three screening assays, as well as the results of binning strategies. The PubChem activity score indicates whether a gene was ‘active’ (designated 2, i.e., a screen hit) or ‘inactive’ (designated 1, i.e., not a screen hit). Samples are defined by siRNA catalogue number (Thermo Fisher) and Entrez Gene ID. Genes included in the seed cluster analysis are indicated with ‘hit’ or ‘null’ depending on whether they were a screen hit or not.

### Data record 2

Secondary deconvolution siRNA screen data are available at PubChem under the accession number AID 743458 (Data Citation 2). Screen-wide raw and normalised data (negative control normalisation, where appropriate) are provided for the three screening assays, as well as the results of binning strategies. PubChem activity score and sample definition are as Data record 1 above.

### Data record 3

Tertiary siRNA screen data are available at PubChem under the accession number AID 743448 (Data Citation 3). Screen-wide raw and normalised data (negative control normalisation and robust *z*-score normalisation, where appropriate) are provided for the three screening assays and four cell lines, as well as the results of binning strategies. PubChem activity score and sample definition are as Data record 1 above.

### Data record 4

miRNA mimic screen data are available at PubChem under the accession number AID 743456 (Data Citation 4). Screen-wide raw and normalised data (negative control normalisation and robust *z*-score normalisation, where appropriate) are provided for the three screening assays, as well as the results of binning strategies. PubChem activity score and sample definition are as Data record 1 above.

### Data record 5

RNAseq data are available at the Gene Expression Omnibus (GEO) under the accession number GSE56788 (Data Citation 5). Individual BAM files are provided for each sample sequenced, as well as the normalised expression matrix generated in Limma-Voom.

## Technical Validation

### RNAi screening

#### Replicate plate reproducibility

**Figure 3:**
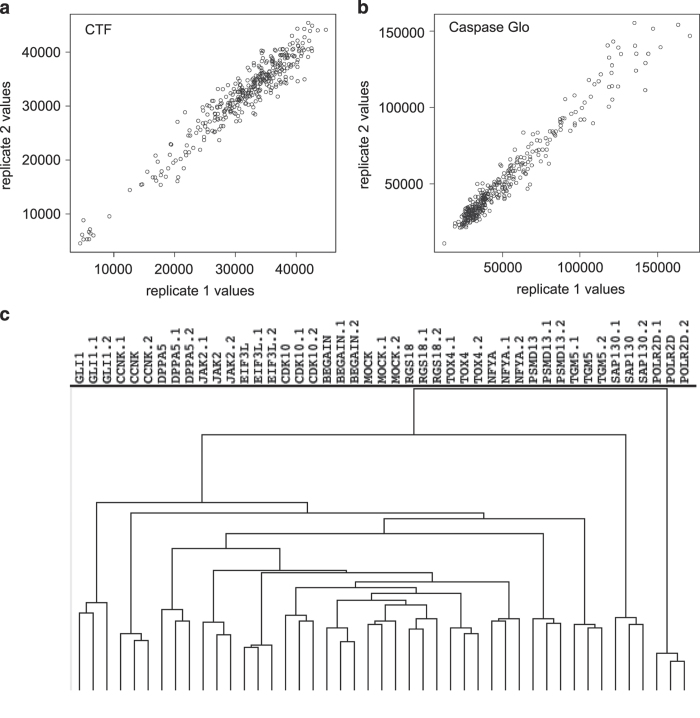
Replicate reproducibility. Example replicate plate reproducibility plots (library plate 12006) for (**a**) CTF and (**b**) Caspase-Glo 3/7 assays. The Pearson Correlation Co-efficient for the two assays is 0.96 and 0.98 respectively. The Spearman’s Rank Correlation Coefficient is 0.93 and 0.95 respectively. Raw fluorescence units (**a**) or raw luminescence units (**b**) are plotted for all library samples (i.e., no controls) for duplicate assay plates. (**c**) Unsupervised hierarchical clustering was conducted for all siRNA samples and mock transfection control, using average linkage and a standard deviation cut-off of 0.75. Triplicate samples cluster together in all cases, indicating a high level of replicate reproducibility. Hierarchical clustering was performed by Cluster and viewed in TreeView.

Replicate plate reproducibility was calculated for each set of duplicate plus-drug plates for both the CTF and Caspase-Glo 3/7 assays using the Pearson Correlation Co-efficient and Spearman’s Rank Correlation Co-efficient. Example dot plots for one set of duplicate plates is presented in Figures 3a and b. Acceptable replicate reproducibility was defined as a Pearson Correlation Co-efficient, *r*≥0.70 for CTF and r ≥0.85 for Caspase-Glo 3/7, based on analysis of the entire primary screen (Pearson Correlation Co-efficient mean and standard deviation across primary screen for CTF: 0.86, 0.062 and for Caspase-Glo 3/7: 0.93, 0.025). The CTF correlation co-efficients were consistently lower than those of Caspase-Glo 3/7, most likely due to the higher raw fluorescence values for the majority of CTF samples (and therefore greater magnitude of variability).

#### Control performance

Positive and negative control performance was quantified by the Z′ factor (Table 2), a statistical measure of the dynamic range between positive to negative controls, encompassing the mean and standard deviation of each control which must be at least three standard deviations from each other^17^. Generally, an acceptable Z′ factor for an RNAi screen is ≥0.3. All plates performed very well for CTF controls, with an average mock/siPLK1 Z′ of 0.62 and none lower than 0.19. Caspase-Glo 3/7 controls showed variable Z′ factors across the screen. Therefore, to determine if the Caspase-Glo 3/7 controls were reporting as expected, the Z′ factor in combination with fold change to mock were assessed (Table 2). Provided the fold change of siJAK2 to mock transfection was maintained above 2-fold, the plate was incorporated into the screen. *JAK2* was found to be a moderate strength positive control as the screen identified many siRNAs that co-operated with vorinostat to a far greater extent than siJAK2. Poor Z′ factors in the Caspase-Glo 3/7 assay were also due to the high sensitivity of the assay (compared to CTF) and therefore larger variability within control wells. There was no correlation between plates with low siJAK2 fold change to mock and low Z′ factor, indicating that it was the variability, not magnitude of fold change that resulted in a poor Z′ factor.

#### Primary screen identified known candidates

HCT116-VR cells harbour mutant *KRAS* and, as expected, *KRAS* knockdown was found to be lethal in the minus-drug arm of the primary screen. *JAK2* and *BCLXL* both achieved low CTF robust *z*-scores (*z*=−3.67 and *z*=−3.07 respectively), which confirmed the ability of the screening methodology to identify known genes involved in vorinostat resistance.

#### Biological reproducibility across screening experiments

It was not possible to perform the primary screen in multiple biological replicates, due to the cost of the Apo-Live Glo reagent, therefore a small subset of targets were evaluated in a second experiment. Six of the highest Caspase-Glo 3/7 scoring SMARTpools from the primary screen were retested at the same time as the deconvolution screen to give an indication of reproducibility between biological experiments. Although the Caspase-Glo 3/7 dynamic range of the secondary screen was lower than that of the primary screen (both controls and samples), all 6 SMARTpools robustly reproduced confirming the validity of screening in single biological replicate (Table 3).

#### Numbers of gene validating in the deconvolution screen

Table 4 summarises the frequency of genes validating with different numbers of duplexes for each assay in the deconvolution screen, with 80% of genes tested validating with at least one siRNA duplex. The percentages of genes validating with different numbers of duplexes follows the trend observed in other studies using Dharmacon siRNA libraries^18,20,26,27^.

### Transcriptome analysis

#### Replicate reproducibility

Unsupervised hierarchical clustering was used to assess replicate sample reproducibility using the normalised expression matrix output from the EdgeR Limma-Voom pipeline. The Pearson correlation (uncentred) similarity metric^28^ was used with two different clustering linkage methods, complete and average linkage, and two gene expression standard deviation (s.d.) cut-offs of 0.75 and 1.00. A total of 2197 genes were analysed using a sd cut-off of 0.75, while 942 genes were analysed using an sd cut-off of 1.00. Triplicate samples for mock and each of the siRNA samples were analysed. In each of the four different clustering conditions triplicate samples for each siRNA clustered together, indicating high replicate reproducibility (Figure 3c). Principal components analysis also showed triplicate samples clustering tightly (data not shown). Interestingly, only a single gene was commonly regulated by knockdown of each of the 14 genes assessed by transcriptomics: PPP1R1B protein phosphatase 1, regulatory (inhibitor) subunit 1B (ENSG00000131771, Entrez 84152), a target not known to interact with HDACi.

#### siRNA on-target gene knockdown

Target gene knockdown in each siRNA sample was assessed (Table 5). The differential expression matrix was used to determine the log_2_ fold change for each siRNA target gene in the knockdown samples compared to the mock samples. This data confirmed that for the 11 vorinostat-resistance candidates whose expression was detected, highly efficient target gene knockdown was achieved. Only NFYA was not in the 10 most downregulated genes following knockdown, but still showed a log_2_ fold change of −1.37, which correlated with knockdown of 88% by qRT-PCR (data not shown). For completeness, raw read counts were also investigated for each of the vorinostat-resistance candidates to confirm results from the differential gene expression analysis (note: this analysis was conducted before normalisation).

This RNAseq analysis on mock transfected cells provided valuable insight into what genes were expressed and not expressed in the HCT116-VR cells. This information could have fed in earlier to eliminate the non-expressed genes from our final hit list. RGS18, TGM5 and DPPA5 passed through our stringent validation but can now be excluded as they are acting as off target siRNAs. It is now commonplace in our facility to conduct expression analysis prior to conducting the siRNA deconvolution screen so that non-expressed genes are excluded from further analysis.

## Usage Notes

siRNA and miRNA screening data (Data records 1-4) are provided for users to be able to apply their own normalisation strategies and thresholds for changes in viability and caspase activation. This study focussed on genes that specifically sensitised to vorinostat-induced cell death, however, the tertiary screen data (106 genes) can be analysed for genes sensitising to both or either of vorinostat and etoposide. Furthermore, genes that were lethal by gene knockdown alone can be investigated for their role in cell survival, with proof of principle being the lethality of *KRAS* knockdown. RNAseq data is provided as raw data (BAM files) for those wishing to apply a particular expression analysis pipeline as well as the normalised expression matrix as a human-readable processed data format.

## Additional information

**How to cite this article:** Falkenberg, K. J. *et al.* Genome-wide functional genomic and transcriptomic analyses for genes regulating sensitivity to vorinostat. *Sci. Data* 1:140017 doi: 10.1038/sdata.2014.17 (2014).

## Supplementary Material



Supplementary Information

## Figures and Tables

**Table 1:** Cell culture and transfection conditions.

	**HCT116-VR**	**SW480**	**SW620**	**LIM1215**
**Growth media**	RPMI 1640	RPMI 1640	RPMI 1640	RPMI 1640
	10% (v/v) FBS	10% (v/v) FBS	10% (v/v) FBS	10% (v/v) FBS
	2.5 g/l glucose			
	1% (v/v) sodium pyruvate			
**No. of cells/384 well**	1,400	1,800	2,500	1,300
**Vol DharmaFECT 2 /384 well**	0.06 μl	0.1 μl	0.1 μl	0.1 μl

**Table 2:** Summary of average Z’ factor and fold change to mock for CTF and Caspase-Glo 3/7 assays across the primary screen.

	**CTF (** ***PLK1*** **/mock)**		**Caspase-Glo 3/7 (** ***JAK2*** **/mock)**	
	**Z’ factor**	**Fold change**	**Z’ factor**	**Fold change**
**Min**	0.19	0.13	−0.97	2.17
**Max**	0.89	0.34	0.85	3.56
**Mean**	0.62	0.19	−0.03	2.89
**s.d.**	0.14	0.04	0.42	0.29

**Table 3:** SMARTpool reproducibility between the primary and secondary siRNA screens for Caspase-Glo 3/7.

**Gene Name**	**Entrez Gene ID**	**Library Plate**	**Primary screen**		**Secondary screen**
			**Fold change**	***z*** **-score**	**Fold change**
*DCUN1D2*	55208	12056	5.87	8.32	4.04
*FANCA*	2175	12006	6.84	9.66	4.30
*SIAHBP1*	22827	12038	7.15	9.61	5.97
*NR1H2*	7376	12021	8.14	9.84	5.01
*LSM14A*	26065	12048	8.66	10.67	4.50
*CNOT1*	23019	12051	8.95	13.48	7.44

**Table 4:** Summary of primary screen Caspase-Glo 3/7 and CTF hits by numbers of validating duplexes in each assay.

**Validation status**	**Primary screen Caspase-Glo 3/7**		**Primary screen CTF**		**All primary screen hits**	
	**No. of hits**	**% Hits**	**No. of hits**	**% Hits**	**No. of hits**	**% Hits**
0/4	39	8	53	11	92	20
1/4	87	19	65	14	152	33
2/4	121	26	16	3	137	29
3/4	47	10	8	2	55	12
4/4	23	5	8	2	31	7
**Total**	**317**	**68**	**150**	**32**	**467**	**100**
Note: total of 467 not 450 as 17 genes were primary screen hits in both assays.						

**Table 5:** Confirming target gene knockdown in siRNA samples.

**siRNA target gene**	**Log**_**2**_ **fold change**	**siRNA rank**	**Mean reads in knockdown samples**	**Mean reads in all other samples**	**Percent reads remaining**
*EIF3L*	−6.28	3	110	6,698	1.6
*TOX4*	−5.47	1	19	870	2.2
*PSMD13*	−5.06	1	88	3,357	2.6
*CCNK*	−5.00	1	43	1,124	3.8
*JAK2*	−4.82	2	5	115	4.1
*SAP130*	−4.81	4	26	739	3.5
*GLI1*	−4.25	1	0	11	0
*CDK10*	−3.15	5	23	185	12.3
*POLR2D*	−3.14	9	112	1,046	10.7
*BEGAIN*	−2.56	5	6	25	25.3
*NFYA*	−1.37	174	193	536	36
*TGM5*	na	na	0	0	0
*RGS18*	na	na	0	0	0
*DPPA5*	na	na	0	0	0
Genes are listed in order of greatest to least fold change. The ‘siRNA rank’ column refers to where the siRNA target gene knockdown sits in relation to all other downregulated genes, i.e., EIF3L was the third most downregulated gene in siEIF3L samples whilst TOX4 was the most downregulated gene in siTOX4 samples.					
na=not applicable as expression not detected.					
