# Supplementary material for: Genome-wide functional genomic and transcriptomic analyses for genes regulating sensitivity to vorinostat
Source: Sci Data. 2014 Jul 8;1:140017. doi: 10.1038/sdata.2014.17 (PMC4322586; doi:10.1038/sdata.2014.17)
Supplement: Supplementary Information [file sdata201417-s2.pdf]

Supplementary Information –

Table of Contents

Supplementary Figure 1 – Comparison between different assay readouts page 2
Supplementary Figure 2 – Control locations on screen plates page 4

Supplementary Figure 1

A

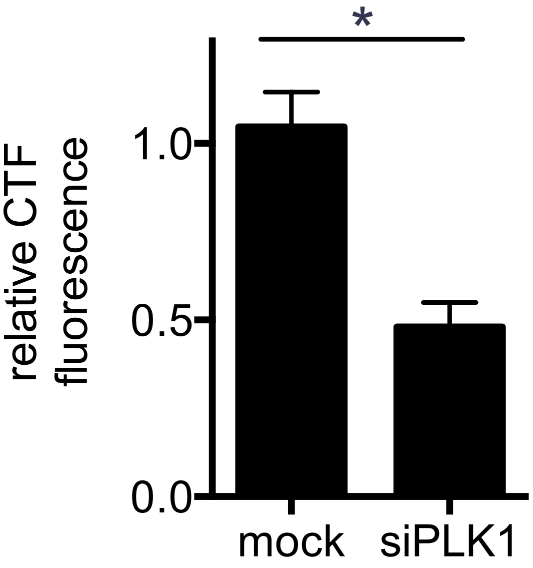

B

	mock	siPLK1
cell count	1637	1080
field count	6	25

C

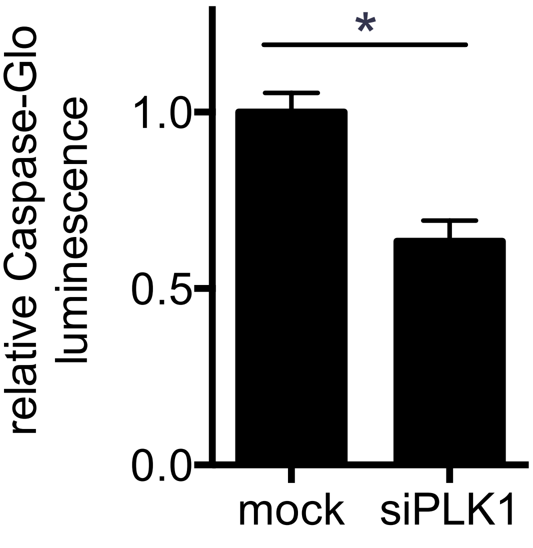

Supplementary Figure 1

Comparison between different assay readouts. At 72 hours post-transfection with siPLK1, cell viability was measured by a) CTF and b) cell counting of DAPI stained nuclei. Reporting both cell count and field number showed a significant difference between a healthy well (≥1500 cells counted in ≤11 fields) and a toxic well (<1500 cells counted in 25 fields). Caspase 3/7 activity (c) shows reduced levels of activated capases 3 and 7 due to the vastly reduced numbers of cells remaining in the wells. * all assays showed a statistical difference between mock and siPLK1 of $p<0.05$.

Supplementary Figure 2

A) Primary SMARTpool screen control well locations

	1	2	3	4	5	6	7	8	9	10	11	12	13	14	15	16	17	18	19	20	21	22	23	24
A		mock																					mock	
B		mock																					mock	
C		JAK2																					JAK2	
D		PLK1																					PLK1	
E		mock																					mock	
F		mock																					mock	
G		JAK2																					JAK2	
H		PLK1																					PLK1	
I		mock																					mock	
J		JAK2																					JAK2	
K		mock																					mock	
L		PLK1																					PLK1	
M		JAK2																					JAK2	
N		PLK1																					PLK1	
O		mock																					mock	
P		mock																					mock	

B) Secondary individual duplex screen control well locations

	1	2	3	4	5	6	7	8	9	10	11	12	13	14	15	16	17	18	19	20	21	22	23	24
A																								
B																								
C							mock					mock					mock					mock		
D							mock					mock					mock					mock		
E							JAK2					JAK2					JAK2					JAK2		
F							mock					mock					mock					mock		
G							PLK1					PLK1					PLK1					PLK1		
H							mock					mock					mock					mock		
I							mock					mock					mock					mock		
J							JAK2					JAK2					JAK2					JAK2		
K							mock					mock					mock					mock		
L							PLK1					PLK1					PLK1					PLK1		
M							mock					mock					mock					mock		
N							mock					mock					mock					mock		
O																								
P																								

Supplementary Figure 2

Plate locations of control wells for the primary SMARTpool (A) and secondary duplex validation (B) screens. Control well locations are indicated for each plate type – mock control (green), siPLK1 (yellow) and siJAK2 (blue). A) For the SMARTpool screen, samples were contained in columns 3-22, indicated in purple. B) For the Duplex validation screen, samples locations are indicated in purple. Duplex 1 siRNAs in columns 3-6, duplex 2 siRNAs in columns 8-1, duplex 3 siRNAs in columns 13-16 and duplex 4 siRNAs in columns 18-21.
